# Role of Toxic Elements in Chronic Kidney Disease

**DOI:** 10.5696/2156-9614-8.20.181202

**Published:** 2018-12-06

**Authors:** Adwalia Fevrier-Paul, Adedamola K. Soyibo, Sylvia Mitchell, Mitko Voutchkov

**Affiliations:** 1 Department of Physics, The University of West Indies, Kingston, Jamaica; 2 Department of Medicine, University Hospital of the West Indies, Kingston, Jamaica; 3 Biotechnology Centre, The University of the West Indies, Kingston, Jamaica

**Keywords:** Complications of chronic kidney disease, minerals, nutrition, kidney function

## Abstract

**Background.:**

The kidney is central to many complex pathways in the body and kidney injury can precipitate multiple negative clinical outcomes. The resultant effect on nutrition and elemental body burden is bi-directional, confounding the very complex pathways that maintain homeostasis. These elemental changes themselves increase the risk of nutritional and biochemical disturbances.

**Objectives.:**

The aim of the present study was to describe how toxic elements interface with complications of chronic kidney disease (CKD).

**Methods.:**

The present review included studies focusing on the molecular mechanisms induced by exposure to elements with known nephrotoxic effects and associated health complications in CKD patients.

**Discussion.:**

Many non-essential elements have nephrotoxic activity. Chronic injury can involve direct tubular damage, activation of mediators of oxidative stress, genetic modifications that predispose poor cardiovascular outcomes, as well as competitive uptake and element mobilization with essential elements, found to be deficient in CKD. Cardiovascular disease is the most common cause of mortality among CKD patients. Oxidative stress, a common denominator of both deficient and excess element body constitution, underlies many pathological derivatives of chronic kidney disease. Bone disorders, hematological dysfunction and dysregulation of acid-base balance are also prevalent in kidney patients. The largest contribution of toxic element body burden results from environmental exposure and lifestyle practices. However, standard medical therapies may also potentiate toxic element accumulation and re-injury of vulnerable tissue.

**Conclusions.:**

For CKD patients, the cumulative effect of toxic elements persists throughout the disease and potentiates complications of CKD. Medical management should be coordinated between a medical team, dietitians and clinical researchers to mitigate those harmful effects.

**Competing Interests.:**

The authors declare no competing financial interests

## Introduction

Multi-element body composition analysis is a common concept for prevention and treatment of disease shared by all brands of medicine—conventional, allopathic and alternative—and a great deal of scientific research has been invested in trace elemental analysis in recognition of that role.^1^

The kidney, a filtration apparatus for blood, is responsible for the removal of toxins and regulation of fluid, molecules and by-products of metabolic processes.^2^ Chronic Kidney Disease (CKD) (*Table 1*), a gradual loss of kidney function or structural abnormality present for three months or longer, with an estimated glomerular rate (eGFR) <60 ml/min in 1.73 m^3^ and/or the presence of albuminuria (>30 mg), and that exerts influence or is influenced by trace elements circulating in the human body.^3^ However, because of its ubiquitous role, a damaged kidney will affect non-linear algorithms, which affect the overall health of kidney patients etc.^4^

**Table 1:**
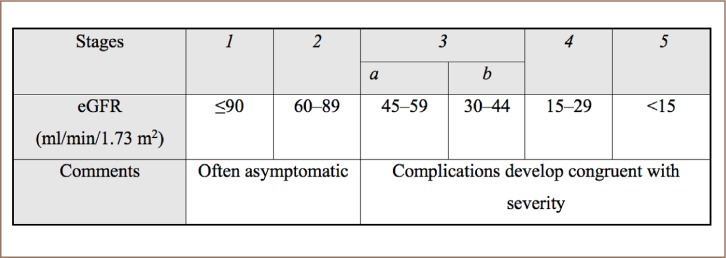
Categories of Chronic Kidney Disease Based on Estimated Glomerular Filtration Rate Abbreviation: eGFR, estimated glomerular function rate

Heavy metal exposure, along with other potential environmental hazards, is a cause of CKD of unknown etiology (CKDu). The concept of CKDu, first used in El Salvador, has been examined across various regions, including Sri Lanka, India, Mexico and the United States. Arsenic exposure has since been linked to CKDu in several studies.^5^

### Toxic element exposure and the effects of accumulation

Humans may be exposed to toxic elements through water, air or consumption of contaminated food products. In Japan, “Itai Itai” disease occurs as a result of cadmium (Cd) poisoning from consumption of Cd-contaminated rice. It is characterized by several health effects, including severe kidney damage.^5^ In Jamaica, Cd levels in kidney and liver tissue samples were highest in study participants from central Jamaica, where environmental and dietary exposure to Cd was also high.^6^ Naturally occurring Cd measured as high as 409 mg/kg in soil.^7^ Animal studies performed in the same area confirmed a ten-fold accumulation of Cd in the kidney compared to samples from non-polluted areas.^8^ In other studies, study subjects with a high level of occupational exposure to lead have shown association with cellular changes, higher creatinine levels and renal injury.^9^ Epidemiological studies investigating mercury exposure and CKD have been less clear.

Other toxic elements such as aluminium (Al), silica (SiO_2_) and strontium (Sr) are eliminated from the body via the kidney. Some of these can be nephrotoxic, and a dysfunctional kidney increases retention of these heavy metals, thus perpetuating further damage.^10^ Apart from the tendency to accumulate within the kidney, hazardous elements are also actively reabsorbed after glomerular filtration via metal transporters, in ways still poorly understood. It is through these mechanisms that some postulate a disruption in the normal re-absorption of essential micro-nutrients from urine. The long half-life of these elements, their affinity to absorb and accumulate within the bone and kidney, disruption of normal hormonal processes and essential trace elemental and ligand interactions are all factors increasing retention within the body and potentiating toxicity in the kidney.^11^,^12^

Not surprisingly, essential elements are also severely altered in CKD. Decreased levels of micronutrients such as zinc, selenium, iron and calcium have been reported by several studies, as a direct result of diminishing kidney function, although the mechanisms underlying those changes vary.^13^,^14^ In addition to causing nutrient deficiencies, CKD may result in overload disorders, a direct result of reduced CKD excretion and interdependent element relationships. Several studies have demonstrated an accumulation of chromium, vanadium, nickel and copper in CKD patients.^15^ Furthermore, medical therapies and dietary restrictions contribute significantly to those changes.

**Abbreviations**
*CKD*: Chronic kidney disease
*CKDu*: Chronic kidney disease of unknown etiology
*DMT-1*: Divalent metal transporter 1
*RAAS*: Renin-angiotensin aldosterone system

Cardiovascular disease and mineral bone disorder rank as the most common complications of severe kidney disease. Poorly controlled kidney disease also escalates dyslipidemia, inflammatory and malnourished states. More severe sequelae such as uremic bleeding, pericarditis, uremic neuropathy, thyroid and sexual dysfunction, depression and infection can also occur.^16^ Undesirable elemental trends may play a role in each of these complications and should not be overlooked. The added effects of co-morbid conditions and dialysis and drug therapy which may amplify or nullify observable elemental changes in those patients justify further investigation of the role of trace elements in kidney disease and its major complications.^17–19^ The objective of the present study is to review the research related to toxic elements in kidney function contributing to complications of chronic kidney disease.

### Toxic element effects and complications of CKD

Chronic diseases can be exacerbated by CKD. Examples include cardiovascular disease, bone disorders and oxidative stress.

#### Cardiovascular disease

Cardiovascular mortality is significantly higher in patients with severe kidney disease than in the general population.^20^,^21^ In fact, cardiovascular risk increases from early stages of kidney impairment. Hypertension, a well-established cardiovascular risk factor, can be precipitated by glomerular changes, sodium and fluid dysregulation, all consequences of CKD.^22^ Other cardiovascular risk factors such as anemia, arterial disease, hyperhomocystenemia and prothrombotic factors also carry significant morbidity.^23^,^24^

A number of heavy metals play a role in complications of CKD, such as cardiovascular disease and elevated blood pressure, including lead, cadmium and arsenic. Multiple studies have a shown a positive association of lead to blood pressure and cardiovascular disease.^25^ Nationally representative studies have confirmed these associations despite declining lead levels in the general population.^11^,^26^,^27^ Lead interferes in a variety of enzymatic processes which correlate negatively with cardiovascular health. Workers subjected to chronic lead exposure showed reduced synthesis and renal excretion of 6-keto-prostaglandin factor 1-alpha, a vasodilator and enhanced synthesis and excretion to vasoconstrictors such as thromboxane. Lead may also contribute to anomalies within the renin-angiotensin aldosterone system (RAAS). These exposures, along with the impairment of oxidative phosphorylation, energy production, and lead accumulation, promote a hypertensive state. Enzymes studies have also shown that lead toxicity impedes δ-aminolevulinic acid dehydratase, ferrochelatase and coporphyrin oxidase activity, enzymes in the heme synthesis pathway, and can precipitate sideroblastic anemia.^26^,^28^

Like lead, cadmium has been implicated in hypertensive patients with ischemic heart disease.^29^ Several mechanisms have been postulated to be responsible for these changes, including dysregulation of vasoactive mediators such as nitric oxide, direct interference in calcium metabolism which controls smooth muscle wall contraction of blood vessels and endothelial cells and/or hormonal disruption of the RAAS system, and the sympathetic nervous system which results in the cumulative effect of elevated blood pressure.^28^,^30^

With regard to arsenic, few studies have been carried out to investigate its associations with hypertension, and thus the positive association with blood pressure was more ambivalent.^31^ However, animal studies demonstrate increasing vasoconstriction and arrhythmia leading to cardiovascular dysfunction with arsenic exposure.^32^

#### Bone disorders

Several components which maintain mineral and bone metabolism including calcium, phosphorus, vitamin D, parathyroid hormone and fibroblast growth factors are regulated by the kidney.^33^,^34^ Kidney impairment often precedes mineral imbalances, for which the body attempts to compensate in a variety of ways. An example of this, Vitamin D, is primarily activated in the kidney, and thus is depleted in CKD.^35^,^36^ Normally, the bone and gut attempt to maintain serum calcium levels by increasing calcium bone resorption and intestinal absorption respectively.^37^ These compensatory mechanisms, however, cannot sustain demands set by long-term irreversible kidney damage, without significant distortion of bone mineralisation, and by extension, the compromise of its structural integrity.^33^,^38^ Less beneficial minerals or potential hazardous elements will attempt to occupy the sites left void by these mineral changes.

Several studies suggest that strontium reduces the risk of bone fractures and increases bone mineral density.^39–41^ These findings were significant, if somewhat controversial, prompting the practice of strontium supplementation in osteoporosis treatment and prevention. In 2004, the European Medicines Agency approved the use of strontium ranelate for osteoporotic treatment in high risk groups. In 2014, however, the agency limited its recommendation to patients resistant to other forms of drug therapy, due to significant cardiac adverse effects. This approach is still met with skepticism by other food and drug regulatory bodies, and subsequently, manufacturers have discontinued production due to low usage.^42–44^ In animal studies, low dose administration provided a structural advantage, while high exposure resulted in defective bone structure. Currently, its usage is prohibited in patients with severe kidney disease citing a proclivity for strontium accumulation in dialysis patients. An increase in nerve related disorders and bone disease have been linked to this accumulation. Patients prone to low calcium dietary intake are also found to be susceptible to strontium-induced osteomalacia.^45^,^46^

Increased cadmium exposure also increases the risk of low bone mineral density. Cadmium competes with calcium entry and its role in inhibiting vitamin D activation depresses bone remodeling and interferes with the maintenance of bone structural integrity. Moreover, cadmium-induced stimulation of cellular signaling pathways may result in bone resorption and accentuate bone weakening already occurring secondary to renal defects.^47^,^48^ Like cadmium, lead also inhibits the conversion of vitamin D to its active form.

#### Oxidative stress

Oxidative stress is at the nucleus of many molecular dictums that destroy tissue locally, and vessel and organ targets systemically. Toxic element-induced oxidative stress includes the increased production of reactive oxidative species, depletion of anti-oxidative scavengers and indiscriminate binding to resident proteins that disrupt normal physiochemistry within the tissue.^4^,^49^,^50^ Chronic kidney disease, itself, is veritably pro-oxidant and the pluricausal effect of reactive species is responsible for many CKD complications including cardiovascular disease. Evidence shows prominent roles for oxidizing mediators for atherosclerosis, congestive heart failure and thrombogenic events. Of these mechanisms, activation of the Janus kinase and signal activator and transducer of transcription proteins pathway precipitates various signaling mediators and inflammatory processes often associated with kidney injury and progression of disease, including diabetic nephropathy. A uremic environment engendered in severe CKD dismantles the anti-oxidative mechanisms that provide balance to avidly reactive species. Elevated stress markers (a consequence of poor kidney excretion and toxin removal), lipid peroxidation and cytokine-mediated reactive species (created with dialyzer membrane interactions) precede developments of atherosclerosis, ischemic heart disease and stroke. Some evidence shows that mercury chloride-, cadmium chloride- and arsenic-induced effects have contributed in those processes.^20^,^51–54^ High reactive oxidative levels have been observed with lead exposure.^55^

Other oxidative changes are speculated to be responsible for lead-induced chronic interstitial nephritis, cadmium-activated apoptosis and disruption of trans-epithelial junctional integrity in the tubule, silicon triggers for auto-antibody production and the injurious effect of arsenic on the proximal convoluted tubule.^4^,^56^,^57^ Historically, aluminum has been known to potentiate oxidative stress; however, the use of aluminum-based phosphate binders and dialysate have been phased out because of its role in bone de-mineralization and toxicity.^33^

### Metal-ion transporters, exposure and chronic kidney disease risk

Competitive entry between toxic and essential trace elements can also result in trace element deficiencies.^56^,^58^ Inter-relationships between lead-iron, lead-calcium, lead-vitamin D, arsenic-phosphate, mercury-selenium and thallium-potassium have been reported as contributors to micronutrient deficiencies.^56^

Often, heavy metal exchange is not distinguished by biological role, but rather by molecular affinity and the chemical properties of the element in question. For example, divalent metal transporter 1 (DMT-1) preferentially mobilizes iron past the duodenum, liver, red blood cells and proximal convoluted tubule in the kidney. However, DMT-1 also binds other divalent ions, including zinc, copper, manganese, cobalt, nickel, lead and cadmium. Low circulating iron stores induces over-expression of DMT-1, thereby increasing absorption of other divalent ions including cadmium and lead. Zinc deficiency also accentuates lead and cadmium DMT-1 uptake and metallothionein binding. In addition, ZIP-8, a metal-ion transporter like DMT-1, non-selectively binds other divalent metal species dependent on iron and zinc availability. As such, in chronic disease conditions as CKD, decreased elemental levels potentiate exposure to toxic heavy metals and their harmful effects.^4^,^9^ Specialized proteins such as glucose transporter (GLUT1, GLUT5), integral membrane proteins which handle water movement across membrane (aquaporins), organic anion transporting peptide (OATP2B1), drug efflux transporters (MRP2, MATE1), among others, have been identified as docking stations for otherwise non-essential heavy metals. That elemental uptake can occur at the renal tubular level heightens chronic toxic exposure.^9^

Substantial scientific evidence suggests that cadmium interferes with the transport of calcium, zinc, copper and selenium across intestinal barriers. It also has a specific affinity to divalent metal transporters, metallothioneins, albumin, and renal sodium-amino acid cotransporters, which normally facilitate movement of different essential micronutrients across membranes in the intestines, red blood cells, liver and the kidney. As a result, it has been implicated as an oxidative stressor by disruptive binding to glutathione, activation of calcium-depleted enzymes resulting in cell death, and the upregulation of specialized proteins, thereby increasing paracellular cadmium reabsorption across renal tubular epithelial cells. On cadmium administration, animal studies have reported glycosuria, phosphaturia and aminoaciduria, hypertension and renal damage. Mercury, like cadmium, also complexes readily with cysteine residues supplanting zinc and reducing reabsorption.^4^,^56^,^58^,^59^

Nutrition plays an integral role in mitigating exposure effects as implied with increased DMT-1 activity in low iron stores. Animal studies show that adequate calcium balance limits lead accumulation, an effect not seen in low calcium states. Lead also competes with calcium uptake in mitochondria, ganglion cells and at the intestinal level. Additionally, in severe calcium deficiency, vitamin D levels were significantly decreased by elevated lead ingestion.^60^,^61^ In CKD patients already prone to vitamin D deficiency, high mortality risk follows low calcium levels. Supporting evidence shows that administration of zinc, iron, selenium and copper corrects cadmium- and lead-mediated toxic effects and precludes increased cadmium reabsorption in the renal tubules.^56^,^58^

## Conclusions

Chronic kidney disease is a multifactorial disease, affected in part by endogenous and exogenous processes influencing elemental changes, which may potentiate disease progression. Chronic kidney disease complications such as cardiovascular disease, bone demineralization and oxidative stress can also exacerbate or be precipitated by elemental imbalances, causing further deterioration of patient quality of life and increasing mortality risk. Dietary choices and medical therapies contribute to these aberrations and must be deliberately managed in vulnerable populations. Essential mineral excesses, as well as deficiencies and chronic level exposure to toxic elements cause considerable health risk and play a pivotal role in kidney disease complications, aggravating or resulting from damage to the kidney. The approach to medical therapies such as dialysis should be a comprehensive effort, coordinated between a medical team, dietitians and clinical researchers to mitigate the harmful effects of CKD.
